# Supplementary material for: Qualitative analysis from the social referents perspective of the multidimensional construct of schoolchildren’s motor competence
Source: PLoS One. 2022 Dec 19;17(12):e0275196. doi: 10.1371/journal.pone.0275196 (PMC9762582; doi:10.1371/journal.pone.0275196)
Supplement: S1 File — (DOC) [file pone.0275196.s001.doc]

**Classification tree**

1. Motor level:
   1. Coordination and balance:
      1. Motor-perceptual capacities: they refer to those perceptions in which the optimum performance of infant motor conducts is evaluated by the coordination capacities that body knowledge implies (balance, coordination, body outline, laterality).
   2. Physical capacities:
      1. Conditional capacities: they refer to those perceptions in which the optimum performance of infant motor conducts is evaluated by basic physical capacities (speed, force, resistance, flexibility).
   3. Basic and specific motor skills:
      1. Basic motor skills: they refer to those perceptions of good motor conduct being considered to be performed by means of fundamental motor patterns based on carrying out movements and jumps using upper and lower limbs, and not using objects.
      2. Specific motor skills: they refer to those perceptions of good motor conduct being considered to be performed by means of motor patterns based on carrying out hitting and controlling objects using different materials (rackets, bats, etc.), and also guiding, catching or throwing mobile elements using limbs.
2. Cognitive level:
   1. Perceived motor skill:
      1. Perception of the fine motor skill: it refers to the perception of a schoolchild’s consideration of having a good motor skill based on the motor actions related to handling objects and mobile elements when conforming to the motor skill concept.
      2. Perception of the gross motor skill: it refers to the perception of a schoolchild’s consideration of having a good motor skill based on the motor actions related to using one’s body (no objects and mobile elements) when conforming to the motor skill concept.
3. Affective-emotional level:
   1. Self-esteem:
      1. Positively evaluating one’s image and social relationship: perception of the role and influence that positive feelings play and have on schoolchildren’s (considering body image well, positively judging their general conduct or in peer relationships) while performing school motor skills.
   2. Anxiety:
      1. Nervous attitudes and insecurities: perception of the role and influence that negative feelings have on schoolchildren (nervousness, fear of making mistakes, or losing both individually and as a peer group) play and have while performing school motor skills.
   3. Commitment to learning:
      1. Interest and positive implication: they refer to evaluating the attitudes that schoolchildren take to positive conducts to learning and performing physical activities at school and in other closely related school environments (federations, associations, clubs, etc.).
      2. Negative conducts and lack of interest: they refer to evaluating the attitudes that schoolchildren take in terms of negative conducts and lack of interest in learning and performing physical activity at school and in other closely related school environments (federations, associations, clubs, etc.).
4. Social level:
   1. Promotion agents in the family and friendships:
      1. Family members: social support actions and messages from mothers, fathers and grandparents that promote contexts of performing physical activity and peer relationships.
      2. Friendships: social support actions and messages from classmates and friends from school, and from other club members and friends, that promote contexts of performing physical activity and peer relationships.
   2. Promotion agents in the school environment:
      1. Physical Education teachers: actions and messages from teachers who specialise in the Physical Education area that promote contexts of performing physical activity and peer relationships.
      2. Other teachers and management teams: teachers who teach in other Primary Education areas (except Physical Education) and management team members (counsellors, heads of study, headmasters/headmistresses).
   3. Promotion agents outside school:
      1. Sport trainers/monitors: actions and messages from trainers/monitors of sport clubs, associations or sport schools that promote contexts of performing physical activity and peer relationships.
   4. Promotion agents in the institutional domain:
      1. Institutions and public administrations: promotional actions and messages from public institutions and public administrations related to sport, education and culture from the government through programmes and plans that favour performing physical activity in different contexts.
