# Supplementary material for: Qualitative analysis from the social referents perspective of the multidimensional construct of schoolchildren’s motor competence
Source: PLoS One. 2022 Dec 19;17(12):e0275196. doi: 10.1371/journal.pone.0275196 (PMC9762582; doi:10.1371/journal.pone.0275196)
Supplement: S2 File — (DOC) [file pone.0275196.s002.doc]

**Semistructured interview script and discussion group**

1. What motor skills do you think are more related to good movements while schoolchildren perform any physical activity? Why?
2. What actions do you think a child relates to being motor-competent, handling objects like balls, rackets, or only those that use the body? Why?
3. What kind of feelings or sensations does a boy/girl show *a priori* when performing motor actions with other classmates?
4. How do you perceive the importance of self-concept and self-esteem for performing motor actions?
5. In psychological terms, what effect do you think that a child performing motor actions in a known environment with other classmates can have?
6. What type of emotional barriers or difficulties can a child encounter when performing motor actions in their usual context?
7. What conduct do schoolchildren show to perform physical activity in knowledge areas during teaching hours?
8. What engagement do schoolchildren show to perform out-of-school physical activity both at school and outside school?
9. Which people do you think are responsible for promoting schoolchildren’s practice of physical activity bearing in mind the social and health benefits that it provides?
10. Which people in their close context do you think promote children performing physical activity to a greater extent?
