# Supplementary material for: Qualitative analysis from the social referents perspective of the multidimensional construct of schoolchildren’s motor competence
Source: PLoS One. 2022 Dec 19;17(12):e0275196. doi: 10.1371/journal.pone.0275196 (PMC9762582; doi:10.1371/journal.pone.0275196)
Supplement: S3 File — (DOC) [file pone.0275196.s003.doc]

**Consolidated criteria for reporting qualitative studies (COREQ): 32-item checklist**

| **No** | **Item** | **Guide questions/description** |
| --- | --- | --- |
| **Domain 1: Research team and reflexivity** |  |  |
| Personal Characteristics |  |  |
| 1. | Interviewer/facilitator | Which author/s conducted the interview or focus group?  First author. The second and third author was not involved in data collection. |
| 2. | Credentials | What were the researcher's credentials? *E.g. PhD, MD*  The first author is a PhD candidate. The second and third authors are PhD. |
| 3. | Occupation | What was their occupation at the time of the study?  First author: Professor, second author: Associate Professor, third author: Associate Professor. |
| 4. | Gender | Was the researcher male or female?  All the authors are men. |
| 5. | Experience and training | What experience or training did the researcher have?  The authors have 3, 10, and 13 years of experience as researchers. The first author is a PhD student. The second author is a Physical Education researcher. The third author is a Developmental Psychology and Neuroeducation researcher and methodologist. |
| Relationship with participants |  |  |
| 6. | Relationship established | Was a relationship established prior to study commencement?  Participants met the first author for the first time when the data collection began. |
| 7. | Participant knowledge of the interviewer | What did the participants know about the researcher? e*.g. personal goals, reasons for doing the research*  The participants knew that the authors were researchers from a local university. The informed consent forms explained the general study purposes. |
| 8. | Interviewer characteristics | What characteristics were reported about the interviewer/facilitator?  Participants knew that the first author was a PhD student who was conducted one research study for his thesis. |
| **Domain 2: study design** |  |  |
| Theoretical framework |  |  |
| 9. | Methodological orientation and Theory | What methodological orientation was stated to underpin the study?  Content analysis was chosen as methodological orientation. |
| Participant selection |  |  |
| 10. | Sampling | How were participants selected?  Sampling was done following a convenience sampling process that considered several criteria: gender-parental role (Woman-Mother/Man-Father), lessons course (4º, 5º or 6º grade), type of educational institution (State/Private), and availability and willingness to participate. |
| 11. | Method of approach | How were participants approached? e*.g. face-to-face, telephone, mail, email*  An email was firstly sent with an invitation to participate in the study. Next a Google Forms link was forwarded with informed consent and to request data to identify the sample. Discussion group and individual semi-structured interviews were online. |
| 12. | Sample size | How many participants were in the study?  38 family members and 19 teachers took part in the study. |
| 13. | Non-participation | How many people refused to participate or dropped out? Reasons?  There were not participants who dropped out from the study. |
| Setting |  |  |
| 14. | Setting of data collection | Where was the data collected? e*.g. home, clinic, workplace*  The participation was online. |
| 15. | Presence of non-participants | Was anyone else present besides the participants and researchers?  There was no-one else present other than participants and researchers. |
| 16. | Description of sample | What are the important characteristics of the sample? *e.g. demographic data, date*  There were 19 participants in the teacher category (40.47±10.8 years): 8 (41.11%) women and 11 (58.89%) men; 8 (41.11%) belonged to public and 11 (58.89) to private schools. Finally, 7 (36.84%) were teaching in 4^th^ grade, 6 (31.57%) in 5^th^ grade and the last 6 (31.57%), in 6^th^ grade. On the other hand, there were 38 participants in the family members category (42.50±2.83 years), 17 (44.73%) men and 21 (55.26%) women. Family members are equally divided between public and private centres (n=19, 50% in each groupe). By courses, 14 family members (36.84%) belong to 4^th^ grade, 13 (26.31%) to 5^th^ grade and 11 (28.94%) to 6^th^ grade. |
| Data collection |  |  |
| 17. | Interview guide | Were questions, prompts, guides provided by the authors? Was it pilot tested?  The authors prepared a set of questions for the semi-structured interviews and focus groups in advance. It was not pilot-tested. However, was reviewed by an external person to the study, an expert in qualitative methodology and with a professional background related to the Physical Education (PE) area. |
| 18. | Repeat interviews | Were repeat interviews carried out? If yes, how many?  There were no repeat interviews |
| 19. | Audio/visual recording | Did the research use audio or visual recording to collect the data?  All the interviews and discussion groups were audio-recorded and video-recorded to be able to differentiate voices later. |
| 20. | Field notes | Were field notes made during and/or after the interview or focus group?  No, there were not. |
| 21. | Duration | What was the duration of the interviews or focus group?  Focus group interviews took 40 minutes on averages. Individual semi-structured interviews took 25 minutes on average. |
| 22. | Data saturation | Was data saturation discussed?  Yes, it was. |
| 23. | Transcripts returned | Were transcripts returned to participants for comment and/or correction?  No, transcripts were not returned. |
| **Domain 3: analysis and findings** |  |  |
| Data analysis |  |  |
| 24. | Number of data coders | How many data coders coded the data?  Two data coders coded the data. |
| 25. | Description of the coding tree | Did authors provide a description of the coding tree?  Yes, the authors provided a description. |
| 26. | Derivation of themes | Were themes identified in advance or derived from the data?  The analysis was deductive (to study previously raised problems and elements). The original classification tree was built based on the previously considered concept. |
| 27. | Software | What software, if applicable, was used to manage the data?  The Nvivo software (version 12 Plus, <https://www.qsrinternational.com/nvivo/home>)  was used to analyze all the contents from interviews and focus groups. |
| 28. | Participant checking | Did participants provide feedback on the findings?  No, they did not provide feedback. |
| Reporting |  |  |
| 29. | Quotations presented | Were participant quotations presented to illustrate the themes / findings? Was each quotation identified? e*.g. participant number*  Yes, a real quote from a participant was used in each thesis on all the themes. An in-depth hermeneutical analysis was performed.  Yes, each quotation was identified. The coding used to identify each extract was based on five digits: a descriptor of the analysis group ("FAM" for relatives; "MAE" for teachers), and the number which refers to the specific participant. |
| 30. | Data and findings consistent | Was there consistency between the data presented and the findings?  Yes, an attempt was made to gain consistency through triangulations. |
| 31. | Clarity of major themes | Were major themes clearly presented in the findings?  Yes, the major themes are clearly identified by section headings. |
| 32. | Clarity of minor themes | Is there a description of diverse cases or discussion of minor themes?  Yes, there is. |
