# Supplementary material for: Qualitative analysis from the social referents perspective of the multidimensional construct of schoolchildren’s motor competence
Source: PLoS One. 2022 Dec 19;17(12):e0275196. doi: 10.1371/journal.pone.0275196 (PMC9762582; doi:10.1371/journal.pone.0275196)
Supplement: Attachment — Submitted filename: Letter_Response_Revision_PlosOne_Final_Version.doc [file pone.0275196.s004.doc]

Response to reviewers and Editor

Dear Editor.

It is a pleasure for us to receive the opportunity to improve our manuscript by taking into account the Editor’s useful comments and the Reviewer’s interesting comments.

We would like to thank both the Editor for making the revision of our manuscript possible and the Reviewer for all his/her comments and suggestions, which have undoubtedly improved the writing and quality of the study. Below we list our replies to each comment and suggestion made. Changes are highlighted and included in the manuscript.

**EDITOR´S COMMENTS**

**1. Please ensure that your manuscript meets PLOS ONE's style requirements, including those for file naming. The PLOS ONE style templates can be found at 
https://journals.plos.org/plosone/s/file?id=wjVg/PLOSOne_formatting_sample_main_body.pdf and 
https://journals.plos.org/plosone/s/file?id=ba62/PLOSOne_formatting_sample_title_authors_affiliations.pdf**

*Response*: We have ensured that the revised version meets PLOS ONE’s style requirements.

**2. Please provide additional details regarding participant consent. In the ethics statement in the Methods and online submission information, please ensure that you have specified what type you obtained (for instance, written or verbal, and if verbal, how it was documented and witnessed). If your study included minors, state whether you obtained consent from parents or guardians. If the need for consent was waived by the ethics committee, please include this information.**

*Response*: In the ethics statement in the Methods and the online submission information we have specified what type of consent form we obtained. It was obtained in writing. Likewise in the Data collection procedure section, we have indicated that this informed consent was again recalled verbally at the beginning of both the interviews (family members) and focus groups (teachers). Our study did not include minors.

**3. Please change "female" or "male" to "woman" or "man" as appropriate, when used as a noun (see for instance** [**https://apastyle.apa.org/style-grammar-guidelines/bias-free-language/gender**](https://apastyle.apa.org/style-grammar-guidelines/bias-free-language/gender)**).**

*Response*: We have modified “female” or “male” to “woman” or “man”.

**4. In your Data Availability statement, you have not specified where the minimal data set underlying the results described in your manuscript can be found. PLOS defines a study's minimal data set as the underlying data used to reach the conclusions drawn in the manuscript and any additional data required to replicate the reported study findings in their entirety. All PLOS journals require that the minimal data set be made fully available. For more information about our data policy, please see** [**http://journals.plos.org/plosone/s/data-availability**](http://journals.plos.org/plosone/s/data-availability)**.**

*Response*: We have specified where the dataset and transcripts that underlie the results described in our manuscript can be found. The dataset has been deposited in the Figshare data repository.

**5. Please amend your manuscript to include your abstract after the title page.**

*Response*: We have included the Abstract after the title page.

**Additional Editor Comments:**

**We noticed that limitations of the study, like the low number of participants or potential sources of bias, have not been discussed. Please include a paragraph addressing these concerns.**

*Response*: These concerns have been discussed, and we have also added some methodological aspects regarding saturation.

**REVIEWER’S COMMENTS**

**Reviewer #1: First, I would like to congratulate the authors for the efforts done in the manuscript. I think that it is very interesting and It will be a good starting point for other researchers that focus on this field of motor competence. The paper is well structured although the English grammar must be reviewed. For example, "conducted" instead of "carry out".**

**The introduction is well written but a little short. I miss more information related to previous research motor competence related. Which evidence exist? It would be important to highlight why this study is new and why it is important that is published. What does this study add to the already published before?**

*Response*: We would like to thank the Reviewer for his/her comments, which improved the manuscript. The manuscript has been revised by a native English proofreader before submission. However, a second revision has been conducted (see the certificate below). Likewise, the proposed change has been made.

Regarding the Introduction, information related to motor competence research has been added. In this way, the different variables that define the new model of motor competence and the relations found between them have been shown. With all this, the novelty of this work lies in not only the relations between the variables, but also in the incorporation of social agents into the study because they are considered a key element in the development of motor competence in the Primary Education stage on which this study focuses.

**About the methodology, I have some concerns that I would like to ask to the authors:**

**• Why did you choose 4, 5 and 6 grade?**

*Response*: In the Spanish education system, grades 4, 5 and 6 correspond to 9- to 12-year olds. As stated in the Introduction, previous studies refer to the importance of the perception of schoolchildren’s significant and close people to deal with these children’s motor competence concept at pre-adolescent ages between 9 and 12 years old. Moreover according to previous studies, the stage between 9e and 12 years old is when the greatest motor development occurs from the evolutionary and structural points of view. It is also a time when cognitive and emotional aspects are especially important in personality development, when physical and psychological changes take place which, in turn, transform their biology, behavior and social relationships.

**• Why did you choose an ethnographic approach? Justify.**

*Response*: We agree with the Reviewer that this issue needs clarifying. We have adjusted this concern in the manuscript.

**• How did you check the grade in which the teachers work?**

*Response*: A convenience sampling process was used to set up the sample and age was one of the variables included in the inclusion criteria. Thus when presenting the informed consent form, the participants were asked to indicate the course in which they were taught.

**• How did you create the interview? Was it validated? Was it reviewed by experts?**

*Response*: The entire categorization process (generation of the system of categories at the three indicated levels, together with their definitions; S1 File) was reviewed by an external person, an expert in qualitative methodology with training and experience in the Physical Education area. Once the system of categories was revised, the semistructured interview and discussion group scripts were prepared in an attempt to generate questions that would allow all the indicators (the system’s third level of concreteness) to be agglutinated in the answers provided by the different groups of participants. These questions were also reviewed by the expert in qualitative methodology, whose contributions (change in the order and wording of some questions) were considered. In the S2 File, we present the final version of the script used in the study. All this information has been included in the Data collection procedure section.

**About the results, it would be interesting to show a table with examples of quotations of each subtheme.**

*Response*: Table 3 has been added, which includes the different employed quotations.

**In the discussion section, you mention one quotation of each theme and subtheme. I would recommend using more quotations.**

*Response*: We did not include quotations in the Discussion section. Quotations were included in the Results section, but more references have been included.

**Finally, the conclusions must be written without references, please remove.**

*Response*: The requested change has been made. Thank you very much. Likewise, the references have been included in the Discussion section.

**Moreover, the strengths, limitations and prospective must be changed to the end of discussion.**

*Response*: The requested change has been made by adding the specific comment on the limitations suggested by the journal's Editors.

**And last, when you say "joint collaboration between families and teachers as education community members seems necessary to promote schoolchildren to develop their motor competence both inside and outside schools", please could you make some examples in that sense?**

*Response*: Some examples have been included in the Conclusions section.

As you can see, we have considered all the advice offered and concerns raised by the Editor and Reviewer.

We hope that you receive our change proposals optimistically with a view to publish this manuscript.

Yours faithfully,

The authors.
